# Dynamic factors associated with COVID-19 vaccine uptake in Cameroon between 2021 and 2022

**DOI:** 10.4102/jphia.v15i1.578

**Published:** 2024-10-24

**Authors:** Dora Tchiasso, Patricia Mendjime, Karl Njuwa Fai, Brenda S. Nana Wandji, Francis Yuya, Éric Youm, Amelia M. Stanton, Ismaila Karimu, Lisa M. Bebell, Lucrece Matchim, Bongkiyung D. Buri, Rodrigue Ntone, Cyrille Yonta, Claudric R. Tchame, Rachelle Essaka, Justin B. Eyong, Audrey Ngosso, Herwin Nanda, Robert Nsaibirni, Mark Ndifon, Lucrèce Eteki, Nadia Mandeng, Anne-Cécile Zoung-Kani Bisseck, Modeste T. Koku, Emilienne Epée, Georges-Alain Etoundi Mballa, Shalom Tchocfe Ndoula, Linda Esso, Yap Boum

**Affiliations:** 1 Public Health Emergency Operation Center, Ministry of Public Health, Yaoundé, Cameroon; 2 Epicentre, Yaoundé, Cameroon; 3 Epicentre, Paris, France; 4 Department of Psychological and Brain Sciences, Boston University, Boston, United States of America; 5 Department of Medicine, Massachusetts General Hospital, Boston, United States of America; 6 Havard Medical School, Boston, United States of America; 7 Laboratoire du Lac, Yaoundé, Cameroon; 8 World Health Organisation, Yaoundé, Cameroon; 9 Division of Operational Research in Health, Ministry of Public Health, Yaoundé, Cameroon; 10 Faculty of Medicine and Biomedical Sciences, University of Yaoundé I, Yaoundé, Cameroon; 11 Médecins Sans Frontières Suisse, Yaoundé, Cameroon; 12 Expanded Programme on Immunization, Ministry of Public Health, Yaoundé, Cameroon

**Keywords:** COVID-19, vaccine, hesitancy, heterogeneity, Cameroon

## Abstract

**Background:**

Little is known about attitudes towards COVID-19 vaccination in sub-Saharan Africa, where immunisation coverage is the lowest in the world.

**Aim:**

The study aimed to identify factors associated with COVID-19 vaccine hesitancy and uptake in Cameroon, and assess changes in these factors over a period of time.

**Setting:**

The study was conducted in the ten regions of Cameroon.

**Methods:**

The authors conducted a two-phase cross-sectional survey in the 10 regions of Cameroon, from July 2021 to August 2021 (Phase one) and from August 2022 to September 2022 (Phase two). We analysed reasons for vaccine hesitancy descriptively and used logistic regression to assess factors associated with hesitancy.

**Results:**

Overall, we enrolled 12 109 participants: 6567 (54.23%) in Phase one and 5542 (45.77%) in Phase two. Of these, 8009 (66.14%) were not interested in receiving the COVID-19 vaccine (*n* = 4176 in Phase one, *n* = 3833 in Phase two). The refusal rate increased significantly in the northern region from 27.00% in Phase 1 to 60.00% in Phase two. The leading contributor to COVID-19 vaccine hesitancy was fear that the vaccine was dangerous, which was significantly associated (95% confidence interval [CI], *p* < 0.05%) with vaccine refusal in both phases. Overall, 32.90% of participants (*n* = 2578) perceived the COVID-19 vaccine to be dangerous. Advanced age, male gender, Muslim religion and low level of education were associated with vaccine acceptance. Participants reported that healthcare workers were the most trusted source of information about the COVID-19 vaccine by 5005 (42.84%) participants.

**Conclusion:**

Despite the investment of the Ministry of Health and its partners in community engagement, focussing on communication about the vaccine efficacy, tolerance and potential adverse events, fear of the vaccine remains high, likely leading to vaccine hesitancy in Cameroon between 2021 and 2022.

**Contribution:**

The study highlight regional variations in COVID-19 vaccine acceptance in Cameroon, with factors age, gender, religion and education influencing willingness to vaccine. Trust in health workers was high, indicating that, tailored, community-led vaccination strategies are key for improving vaccine uptake, not only for COVID-19 but also for future epidemics.

## Introduction

The COVID-19 pandemic has created an unparalleled strain on healthcare systems and economies worldwide, posing a significant concern especially in sub-Saharan Africa.^1^ As case numbers decline, periodic surges continue to occur, leading to more than 704 million confirmed cases and over 7 million deaths globally, as of 03 March 2024.^2,3^ Currently, there is a paucity of validated treatments for this disease, as only one drug (Paxlovid) has been approved by the World Health Organization (WHO) for treatment of patients with mild and moderate forms of COVID-19 who are at high risk of hospitalisation.^4^ The COVID-19 pandemic also led to an unprecedented race for the development of vaccines against severe acute respiratory syndrome coronavirus 2 (SARS-CoV-2).^5^ As of 2020, 183 vaccines were in clinical trials and 199 in pre-clinical studies,^6^ but the quantities of vaccines available remain insufficient to meet different target populations. So far, 10 vaccines have been authorised by the European Medicine Agency.^7^

Despite the availability of various vaccines against COVID-19, ensuring fairness and equitable access for everyone worldwide remains challenging. The ability of the COVID-19 vaccines in controlling the spread of disease relies on the rate of vaccine coverage. Concerns about potential side effects and uncertainties regarding the effectiveness of the vaccines have contributed to COVID-19 vaccine hesitancy.^8^ The Strategic Advisory Group of Experts on Immunization (SAGE) defines vaccine hesitancy as ‘delay in acceptance or refusal of vaccines despite the availability of vaccination services’.^9^ Vaccine hesitancy is identified by the WHO as one of the top 10 threats to global health.^10,11^ Despite major advances in COVID-19 vaccine development and initiatives to ensure equity in access to these vaccines,^12,13^ vaccine hesitancy threatens COVID-19 vaccine uptake.^14^ Several factors influence vaccine acceptance and uptake in a community, among which are complacency, convenience and confidence.^9,15^

Many Africans are hesitant to take the COVID-19 vaccine, and Africa therefore has the lowest vaccine coverage globally. As much as 70.6% of people have received at least one COVID-19 vaccine dose worldwide. However, in low- and middle-income countries (LMICs), the vaccine coverage was only 32.8% as at 03 March 2024.^16^ During this same period, a total of 4.88 million vaccine doses were administered in Cameroon, 3.76 million persons were vaccinated with at least one dose, and 3.19 million persons were fully vaccinated.^2^ Vaccination coverage for at least one dose of COVID-19 was 26.7% for an eligible population (18 years and above) of 13.75 million people,^17^ indicating very low uptake in Cameroon. In Cameroon, COVID-19 immunisation was integrated into the national strategy in April 2021, with Covishield (AstraZeneca, British-Swedish company) followed by Sinopharm BIBP (Sinopham Co. Ltd., China) and then Jcovden (Janssen Pharmaceuticals, Lieden, the Netherlands).^17^

In 2021, Dinga et al. reported high levels of COVID-19 vaccine hesitancy in Cameroon (84.60%).^15^ Attitudes towards the pharmaceutical industry, the trustworthiness and origin of vaccine, and cost considerations may influence vaccine hesitancy. Cameroonians may have more trust in clinical trials of COVID-19 vaccines that involve African scientists than in clinical trials that are based in on other settings^18^. This study, however, used a small sample size, and data collection was done online; therefore, the study population was not evenly distributed nationwide. Arce et al. recently analysed vaccine acceptance and hesitancy in LMICs worldwide, including 13 African countries,^19^ but Cameroon was not included. Because of a lack of information on COVID-19 vaccine hesitancy, and its importance in promoting community engagement thereby potentially increasing vaccine coverage, we sought to identify factors associated with the vaccine hesitancy and its evolution over time in Cameroon.

## Research methods and design

### Study design

We conducted a two-phase nationwide cross-sectional, community-based, descriptive study from 27 July 2021 to 31 August 2021, and from 25 August 2022 to 15 September 2022. The national surveillance data were used to divide the regions into high- and low-transmission strata; high-transmission stratum included regions with the highest reported attack rates of ≥ 0.5 per 1000 individuals (Central, East, Littoral, South and West regions), whereas low-transmission stratum included the five regions with reported attack rates of < 0.5 per 1000 individuals (Adamawa, Far North, North, South-west, North-west regions). We selected 198 clusters in the capital cities using the Epicentre Geo-sampler (https://apps.msf.fr/epiGeoSampler) to ensure the selection of roof-tops and to exclude non-residential areas in both the phases. We uploaded Global Positioning System (GPS) coordinates for all points onto tablets, and the teams used the OsmAnd V.3.9 (OpenStreetMap Navigation Direction: Release 3.9.) application to identify and navigate to each point, which represented the first household within a cluster.

All selected households were invited to participate in the vaccine hesitancy survey. If a selected household was not visited for any reason whatsoever, it was documented, and the next household was selected. Refusals and households where no one were at home during the visits were documented and replaced, and the next household would be selected using the systematic procedure described above.

Ten households per cluster in the high-transmission stratum and five households per cluster in the low-transmission stratum were selected using the Epicentre Geo-sampler. This difference in sample selection was to ensure that at least 1033 participants in the high-transmission stratum and 291 participants in the low-transmission stratum are enrolled. After written consent or assent was obtained, participants aged 18 years and above were interviewed. It should be noted that no vaccine was administered or proposed to participants, and the survey was done only using a questionnaire. Data collection was done using KoBo Collect V2021.2.4 (Open-source Android app for collecting survey data: release 2.4) in Phase one and REDCap mobile app (Research Electronic Data Capture) in Phase two. The data collection tool was changed in Phase two because REDCap was better suited for the survey, offering enhanced ease of query generation for data quality control.

### Study setting

The study area was all 10 regions (189 health districts) in Cameroon.

### Study population

People aged 18 years and above who provided written consent were enrolled in the study. Key exclusion criteria were those who refused to give written informed consent and those unable to properly complete the questionnaire.

### Data collection

Socio-demographic data including gender, age, marital status, educational level, profession and religion were collected to assess heterogeneity. Variables of interest were COVID-19 vaccine hesitancy, reasons for vaccine hesitancy and trusted sources of information about vaccination.

### Data analysis

Data were exported to R V.4.1.2 (A language and environment for statistical computing, Vienna, Austria. http://www.R-project.org/) for cleaning and statistical analysis. The data were summarised in frequency tables (categorical variables) and via summary statistics (quantitative variables). The study population was divided into the following age categories: 18–28 years, 29–39 years, 40–50 years and 50+ years. We used univariate descriptive analyses to assess levels of vaccine hesitancy and a binary logistic regression model to identify factors associated with vaccine hesitancy. Predictor variables included religion, profession, age, educational level and marital status. *P*-value < 0.05 was considered statistically significant, and odds ratios (OR) at 95% confidence interval (CI) were used to indicate the strength of associations.

### Ethical considerations

Ethical approval to conduct this study was obtained from the National Ethics Committee for Research in Human Health of the Ministry of Public Health (No. 2021/07/1371/CE/CNERSH/SP). The study was conducted according to the guidelines of the Declaration of Helsinki (19). Written informed consent was obtained from all subjects involved in the study.

## Results

### Participants’ characteristics: Phase one and two

A total of 6567 (54.23%) participants in Phase 1 and 5542 (45.77%) participants in Phase 2 from 10 regions (189 health districts in Cameroon) were enrolled in this study. The median age was 36 years (interquartile range [IQR]: 28–50 years) and ranged from 18 years and 100 years old. The 29–39 years age group was the most represented, with 30.61% (*n* = 3707) participants. Females were the majority of the sample 52.20% (*n* = 6321). Most participants had secondary level education 43.55% (*n* = 5274) and 9.19% (*n* = 1113) had no education (Table 1). Across study Phases, 69.71% (*n* = 8442) of participants were employed (Table 1). Most of the sample (67.14%, *n* = 8131) identified as Christian. The Littoral (28.84%, *n* = 3492), Center (28.52%, *n* = 3453), and North (8.59%, *n* = 1041) regions were the regions with most participants.

**TABLE 1:** Socio-demographic characteristics of participants: Phase one and two (*N* = 12 109).

Characteristics	*n*	%
**Age categories (in years)**		
18–28	3203	26.45
29–39	3707	30.61
40–50	2496	20.61
50+	2703	22.32
**Gender**		
Female	6321	52.20
Male	574	47.76
Missing values	4	0.03
**Level of education**		
None	1113	9.19
Primary	2391	19.74
Secondary	5274	43.55
Higher	3327	27.47
Missing values	4	0.03
**Occupation**		
Employed	8442	69.71
Unemployed	2425	20.20
Student	1123	9.36
Missing values	119	0.98
**Religions**		
Christian	8131	67.14
Muslim	2604	21.50
Other	846	6.98
No answer	388	3.20
Missing values	140	1.15
**Marital status**		
In a partnership	6552	54.11
Single	5317	43.91
Prefer not to say	99	0.83
Missing values	141	1.16
**Regions**		
Adamawa	762	6.47
Center	3453	28.52
East	417	3.54
Extreme North	864	7.14
Littoral	3492	28.84
North	1041	8.59
North-west	507	4.31
West	580	4.93
South	258	2.19
South-west	389	3.30
Missing values	346	2.86

### Participants’ characteristics and COVID-19 vaccine hesitance in Phase one and two

Between 2021 and 2022, 4176 (63.59%, 95% CI: 62.41–64.75) and 3883 (70.06%, 95% CI: 68.84–71.27) participants, respectively, were not willing to receive the COVID-19 vaccine (Table 2). The 29–39-year-old age group was the least likely to accept the COVID-19 vaccine in both Phases, with 1312 (32.56%, 95% CI: 19.01–20.97) participants in Phase one and 1200 (31.35%, 95% CI: 20.57–22.76) participants in Phase two. Most participants who did not want the COVID-19 vaccine in Phase one were women (2 547, 61.00%, 95% CI: 37.60–39.97), while the majority in Phase two were men (1959, 51.18%, 95% CI: 34.15–36.68). Participants with greater educational level were the least likely to accept COVID-19 vaccination in both Phases. The results for higher education in Phase one and two were: 1085 (26.00%, 95% CI: 15.63–17.44) and 1181 (30.80%, 95% CI: 20.27–22.45); and secondary education in Phase one and two was: 1919 (46.00%, 95% CI: 28.12–30.33) and 1725 (45.00%, 95% CI: 29.9–32.42). In both Phase one and Phase two, most employed participants were not interested in vaccination: Phase one 2547 (62.10%, 95% CI: 37.60–39.97), Phase two 2957 (77.60%, 95% CI: 52.12–54.77). Compared to individuals with other religious identities, Christians were the most reluctant to be vaccinated, with 2857 (69.60%, 95% CI: 42.30–44.71) in Phase one and 2825 (74.3%, 95% CI: 47.39–52.39) in Phase two reporting no vaccination. People who were Muslim had the highest vaccine uptake despite a decrease between 2021 (*n* = 858, 36.20%, 95% CI: 12.25–13.90, *p* < 0.05) and 2022 (*n* = 456, 26.90%, 95% CI: 7.53–8.99, *p* < 0.05).

**TABLE 2:** Socio-demographic characteristics described by COVID-19 vaccine hesitancy: Phase one and two.

Participants willingness to take the vaccine	Hesitancy Phase 1						Hesitancy Phase 2						Total Phase 1 and 2				*p*
	No (*n* = 4176)			Yes (*n* = 2391)			No (*n* = 3833)			Yes (*n* = 1709)			No (*n* = 8009)		Yes (*n* = 4100)		
				
	*n*	%	95% CI	*n*	%	95% CI	*n*	%	95% CI	*n*	%	95% CI	*n*	%	*n*	%	
**Age categories (in years)**	-	-	-	-	-	-	-	-	-	-	-	-	-	-	-	-	< 0.05
18–28	1360	32.560	19.74–21.716	639	26.720	9.02–10.47	876	22.88	14.86–16.79	328	19.23	5.31–6.57	2236	27.930	967	23.610	-
29–39	1312	31.420	19.01–20.97	690	28.850	9.77–11.27	1200	31.35	20.57–22.76	505	29.61	8.37–9.90	2512	31.380	1195	29.170	-
40–50	744	17.820	10.57–12.12	507	21.200	7.08–8.39	850	22.21	14.39–16.31	395	23.16	6.46–7.84	1594	19.920	902	22.020	-
50+	760	18.190	10.80–12.37	555	23.210	7.78–9.15	907	23.70	15.40–17.36	481	28.21	7.95–9.45	1667	20.830	1036	25.290	-
**Gender**	-	-	-	-	-	-	-	-	-	-	-	-	-	-	-	-	< 0.05
Female	2547	61.000	37.60–39.97	1148	48.000	16.56–18.42	1865	48.73	32.46–34.97	756	44.34	12.77–14.59	4412	55.130	1904	46.480	-
Male	1629	39.000	23.76–25.86	1243	52.000	17.98–19.89	1959	51.18	34.15–36.68	948	55.60	16.15–18.15	3588	44.830	2191	53.500	-
Missing values	0	0.000	-	0	0.000	-	3	0.07	-	1	0.06	-	3	0.040	1	0.020	-
**Level of education**	-	-	-	-	-	-	-	-	-	-	-	-	-	-	-	-	< 0.05
None	337	8.070	4.61–5.69	345	14.400	4.69–5.78	259	6.76	4.14–5.27	172	10.10	2.66–3.60	596	7.440	517	12.600	-
Primary	835	20.000	11.91–13.54	604	25.300	8.50–9.92	666	17.40	11.19–12.92	286	16.80	4.29–5.44	1501	18.700	890	21.700	-
Secondary	1919	46.000	28.12–30.33	898	37.600	12.85–14.52	1725	45.00	29.90–32.42	732	42.90	12.34–14.15	3644	45.500	1630	39.800	-
Higher	1085	26.000	15.63–17.44	544	22.800	7.62–8.97	1181	30.80	20.27–22.45	517	30.30	8.59–10.14	2266	28.300	1061	25.900	-
Missing values	0	0.000	-	0	0.000	-	2	0.05	-	2	0.12	-	2	0.020	2	0.050	-
**Occupation**	-	-	-	-	-	-	-	-	-	-	-	-	-	-	-	-	< 0.05
Employed	2547	62.100	37.60–39.97	1534	64.700	22.34–24.40	2957	77.60	52.12–54.77	1404	82.30	24.23–26.54	5504	69.600	2938	72.000	-
Unemployed	1100	26.800	15.85–17.67	638	26.900	9.00–10.4	488	12.80	8.08–9.59	199	11.70	3.12–4.12	1588	20.100	837	20.500	-
Student	455	11.100	6.32–7.56	200	8.430	2.64–0.34	365	9.58	5.95–7.28	103	6.04	1.52–2.25	820	10.400	303	7.430	-
Missing values	74	1.770	-	19	0.790	-	23	0.60	-	3	0.17	-	97	1.210	22	0.540	-
**Religion**	-	-	-	-	-	-	-	-	-	-	-	-	-	-	-	-	< 0.05
Christian	2857	69.600	42.30–44.71	1338	56.400	19.40–21.36	2825	74.30	47.39–52.39	1111	65.50	19.03–21.16	5682	71.900	2449	60.200	-
Muslim	712	17.400	10.10–11.61	858	36.200	12.25–13.90	578	15.20	9.65–11.28	456	26.90	7.53–8.99	1290	16.300	1314	32.300	-
Other	419	10.030	5.80–6.99	142	5.930	1.82–2.54	214	5.58	3.37–4.40	71	4.15	1.00–1.61	633	7.900	213	5.190	-
No answer	114	2.730	1.43–2.08	34	1.420	0.36–0.72	183	4.77	2.85–3.81	57	3.34	0.78–1.33	297	3.710	91	2.220	-
Missing values	74	1.770	-	19	0.770	-	33	0.86	-	14	0.82	-	107	1.340	33	0.800	-
**Marital status**	-	-	-	-	-	-	-	-	-	-	-	-	-	-	-	-	< 0.05
In a partnership	2042	49.800	29.97–32.23	1398	58.900	20.30–22.29	2034	53.60	35.49–3.05	1078	63.60	18.45–20.55	4076	51.600	2476	60.900	-
Single	2040	49.700	29.94–32.19	969	40.900	39.90–15.63	1707	44.90	29.64–32.09	601	35.40	10.05–11.71	3747	47.400	1570	38.600	-
Prefer not to say	20	0.488	0.18–0.46	5	0.211	0.002–0.017	57	1.50	0.78–1.33	17	1.00	0.17–0.49	77	0.975	22	0.541	-
Missing values	74	1.770	-	19	0.770	-	35	0.91	-	13	0.76	-	109	1.360	32	0.780	-

CI, confidence interval.

### Regional variation in COVID-19 vaccine: Phase one and two

**FIGURE 1:**
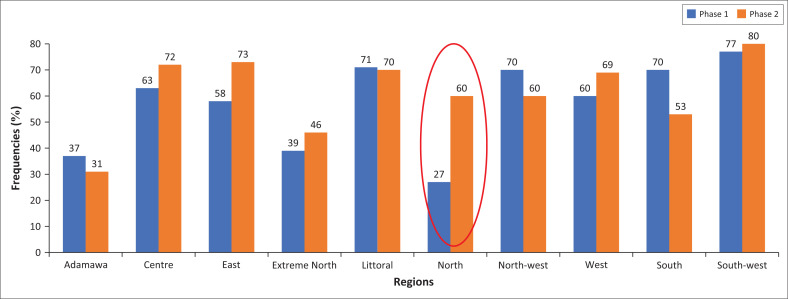
Proportion of people who did not want COVID-19 vaccination, by region in Phase one and two.

Overall, 63.42% of participants were unwilling to receive the COVID-19 vaccine, across both the Phases. The rate increased from Phase one to Phase two in three regions: North (Phase one: 27.00%; Phase two: 60.00%), East (Phase one: 58.00%; Phase two: 73.00%) and Center (Phase one: 63.00%; Phase two: 72.00%), all of which were statistically significant (*p* < 0.05) (see Figure 1).

### Reasons for non-acceptance of the COVID-19 vaccine at the national level

Participants who did not want COVID-19 vaccination were asked to identify the reasons driving their hesitancy (Table 3). For 1328 (33.3%, 95% CI: 31.79–34.74) participants in Phase one and 1250 (32.5%, 95% CI: 31.04–34.03) participants in Phase two, fear that the vaccine was dangerous was the predominant reason for vaccine hesitancy (Table 4). Other reasons that were not specified 876 (21.9%. 95% CI: 20.66) in 2021 and 535 (13.9%, 95% CI: 12.84–15.05) in 2022 were also associated with vaccine hesitancy.

**TABLE 3:** Reasons for vaccine hesitancy at national level for Phase one and two.

Reasons for not wanting to receive the COVID-19 vaccine	Phase 1 (*n* = 3993)			Phase 2 (*n* = 3843)			Total (*N* = 7836)	
	*n*	%	95% CI	*N*	%	95% CI	*n*	%
I am allergic to vaccines	47	1.18	0.86–1.56	57	1.48	1.12–1.91	104	1.33
I am concerned about contracting the virus through vaccination	100	2.50	2.04–3.03	80	2.08	1.65–2.58	180	2.30
I am concerned about the side effects	178	4.46	3.83–5.14	160	4.16	3.55–4.84	338	4.31
I am not concerned about getting COVID-19	13	0.32	0.17–0.55	103	2.68	2.19–3.24	116	1.48
I don’t like needles	128	3.21	2.68–3.79	144	3.75	3.16–4.39	272	3.47
The coronavirus outbreak is not serious	257	6.44	5.69–7.24	161	4.19	3.57–4.87	418	5.33
The vaccine introduces electronic chips in the body	128	3.21	2.68–3.79	249	6.48	5.72–7.30	377	4.81
The vaccine is a mafia	194	4.86	4.21–5.57	291	7.57	6.75–8.45	485	6.19
The vaccine is dangerous	1328	33.30	31.79–34.74	1250	32.50	31.04–34.03	2578	32.90
The vaccine is not effective	169	4.23	3.62–4.90	302	7.86	7.02–8.75	471	6.01
The vaccine was developed too fast	181	4.53	3.90–5.22	170	4.42	3.79–5.12	351	4.48
The vaccines that were sent to Africa are expired	156	3.91	3.32–4.55	98	2.55	2.07–3.09	254	3.24
Vaccines in Africa are different from those in Europe and the United States	132	3.31	2.77–3.90	141	3.67	3.09–4.31	273	3.48
Vaccines were sent to sterilise Africans	106	2.65	2.17–32.01	102	2.65	2.16–3.21	208	2.65
Other	876	21.90	20.66–23.25	535	13.90	12.84–15.05	1411	18.00

Note: *p* < 0.05.

COVID-19, coronavirus disease 2019; CI, confidence interval.

**TABLE 4:** Socio-demographic characteristics associated with COVID-19 vaccine hesitancy using multivariable logistic regression.

Variables	Categories	Phase 1			Phase 2			*p*
		OR	CI 95%	*p*	OR	CI 95%	*p*	
Education	None	2.04	1.70–2.45	< 0.05*	1.52	1.22–1.89	0.001*
	Primary	1.44	1.24–1.67	< 0.05*	0.98	0.82–1.17	0.82
	Secondary	0.93	0.82–1.06	0.21	0.97	0.85–1.11	0.65
	Higher	Ref	-	-	Ref	-	-
Age (in years)	18–28	Ref	-	-	Ref	-	-
	29–39	1.12	0.98–1.28	0.09	1.62	0.95–1.325	-
	40–50	1.45	1.25–1.68	< 0.05*	1.50	1.04–1.47	-
	51–99	1.55	1.35–1.79	< 0.05*	14.75	1.19–1.67	-
Gender	Female	Ref	-	-	Ref	-	-
	Male	1.69	1.53–1.87	< 0.05*	1.19	1.06–1.34	0.002*
Occupation	Employed	Ref	-	-	Ref	-	-
	Unemployed	0.96	0.86–1.08	0.52	0.86	0.72–1.02	0.09
	Student	0.73	0.61–0.87	< 0.05*	0.59	0.47–0.74	< 0.05*
Religion	Christian	Ref	-	-	Ref	-	-
	Muslim	2.57	2.28–2.89	< 0.05*	2.01	1.74–2.31	< 0.05*
	Other	0.72	0.59–0.88	0.001*	0.84	0.64–1.10	0.23
	No answer	0.63	0.43–0.93	0.02*	0.79	0.58–1.07	1.01
	Single	0.69	0.63–0.77	< 0.05*	0.66	0.59–0.75	< 0.05*
Marital status	In a partnership	Ref	-	-	Ref	-	-
Region	Adamawa	Ref	-	-	Ref	-	-
	Center	0.38	0.31–0.47	< 0.05*	0.16	0.12–0.20	< 0.05*
	East	0.53	0.37–0.77	< 0.05*	0.16	0.11–0.22	< 0.05*
	Extreme North	1.26	0.97–1.62	0.07	0.39	0.28–0.54	< 0.05*
	Littoral	0.27	0.21–0.34	< 0.05*	0.18	0.14–0.24	< 0.05*
	North	1.59	1.24–2.05	< 0.05*	0.29	0.22–0.40	< 0.05*
	North-west	0.31	0.22–0.43	< 0.05*	0.29	0.21–0.42	< 0.05*
	South	0.30	0.19–0.47	< 0.05*	0.38	0.25–0.57	< 0.05*
	South-west	0.21	0.15–0.30	< 0.05*	0.95	0.95–0.16	< 0.05*
	West	0.36	0.26–0.50	< 0.05*	0.19	0.14–0.28	< 0.05*

Ref, reference category; OR, odds ratio; CI, confidence interval.

* , Significant *p*-value.

### Factors associated with COVID-19 vaccine uptake and hesitancy

The results of logistic regression demonstrated that participants with no formal education were more likely to accept the COVID-19 vaccine (OR: 2.04, 95% CI: 1.70–2.45; OR: 1.52, 95% CI: 1.22–1.89) relative to those with higher education. Participants aged 50 years and above were more likely to accept COVID-19 vaccine in Phase one (OR: 1.55, 95% CI: 1.35–1.79). In both the Phases, participants aged 40–50 years were the most likely to accept the vaccine though age group differences were not statistically significant (OR: 1.45, 95% CI: 1.25–1.68; OR: 1.50, 95% CI: 1.04–1.47). Male participants were more likely to accept the vaccine than female participants in both Phases (OR: 1.69, 95% CI: 1.53–1.87). Students were less likely to accept COVID-19 vaccine in both Phases compared to employed participants (OR: 0.73, 95% CI: 0.61–0.87; OR: 0.59, 95% CI: 0.47–0.74). Participants from the Centre region, East, Littoral, North-west, South, South-west, West regions were less likely to accept the COVID-19 vaccine *(p <* 0.05). Muslim participants were significantly more likely to accept vaccination than Christian participants in both the Phases (OR: 2.57, 95% CI: 2.28–2.89; OR: 2.01, 95% CI: 1.74–2.31 for Phase one and Phase two, respectively).

### Trusted sources of information about the COVID-19 vaccine

In Phase one, the most trusted sources of information were healthcare workers for 2798 (42.94%) participants and family and/or friends for 792 (12.15%) participants (Table 5). The least trusted sources of information were famous people and/or religious leaders for 210 (3.22%) participants, media for 79 (1.21%) participants, and community leaders for 67 (1.03%) participants. In Phase two, healthcare workers remained the most trusted source of information (Table 5). Other trusted sources of information were family and/or friends for 1157 (22.39%) participants, non-source of information (do not trust any source of information) with 815 (15.77%) participants. In Phase two, the least trusted sources of information were famous people and/or religious leaders for 65 (1.26%) participants, media for just 67 (1.30%) participants; and community leaders for 65 (1.26%) participants.

**TABLE 5:** Trusted source of information about COVID-19 vaccine.

Source of information	Phase 1 (*n* = 6515)		Phase 2 (*n* = 5176)		Total (*N* = 11 682)	
	*n*	%	*n*	%	*N*	%
Family or friends	792	12.15	1157	22.39	1950	16.69
Famous people and/or religious leaders	210	3.22	191	3.69	401	3.43
Government or ministry of health	597	9.16	665	12.84	1262	10.80
Health workers (doctors, nurses, laboratory technicians, etc.)	2798	42.94	2207	42.71	5005	42.84
Media (newspapers, TV, radio, or online groups)	79	1.21	67	1.30	146	1.25
Community leaders	67	1.03	65	1.26	132	1.13
None	1972	30.26	815	15.77	2787	23.6

Note: *p* < 0.05.

TV, television.

## Discussion

Vaccination is crucial to curb the impact of the COVID-19-related mortality. Although vaccination rates are slowly rising in Africa, its acceptance is still a major challenge in Central Africa, including in Cameroon. Our study identified factors associated with nationwide COVID-19 vaccine hesitancy in Cameroon, which were higher than those reported in other African countries during the study period.^20^

Demographic characteristics such as male gender, young age, Christian religion and high level of education were significantly associated with vaccine hesitancy, corroborating other findings in Egypt and Qatar.^21,22^ However, we found that participants with no formal education were more likely to accept COVID-19 vaccination than those with higher education, in contrast to other studies.^21,23^ The reasons for this finding are not entirely clear; though it is possible that individuals with less education may have less access to misinformation spread by social media and may be more likely to trust advice from their leaders.

Vaccine acceptance rates in Cameroon were lower towards the Southern part of the country, compared to the Northern part, a similar regional discrepancy to what has been reported in Congo and in Uganda.^10,24^ One of the hypotheses was that the regions with the highest vaccine acceptance were the regions with a lower Internet connectivity. Those regions would therefore be less exposed to fake news.^24,25^ Muslim participants were more likely to accept the COVID-19 vaccine than Christian participants, which might explain why the highest acceptance rates were found in the northern regions of Cameroon, which also have a higher proportion of people with lower levels of education. These associations were similar in both the study Phases.

Despite higher vaccine acceptance in some regions and groups, COVID-19 vaccination acceptance was overall lower in this study than the average acceptance rate reported in Africa, estimated to be 49% (95% CI: 39–58).^26^ Vaccine acceptance varies widely across countries globally. Worldwide, the highest average vaccine acceptance rates have been reported in China and the United States, followed by European and African countries, with Arab countries reporting the lowest vaccine acceptance rates.^26^ However, vaccine acceptance rates vary with time, with increasing trends as the pandemic evolves.^20^ While socio-demographic characteristics of population and time of survey might account for varying results globally, factors such as the influence of strong community and religious leaders as present in the Northern regions of Cameroon may also influence COVID-19 vaccine acceptance rates.

Our results are consistent with what was reported in a systematic review from 2021 about reasons for vaccine hesitancy worldwide.^20^ However, one major concern reported elsewhere was the price of getting vaccinated,^20^ which was not a major factor contributing to vaccine hesitancy in Cameroon and other sub-Saharan African countries where vaccination is free of charge. However, vaccine-associated costs such as travel expenses or work interruption may also have contributed to low acceptance rates in Cameroon.^27^ In contrast, factors favouring vaccine acceptance in Cameroon appear to include communication with trusted sources including health workers, similar to what has been reported elsewhere.^20^

Low COVID-19 vaccine acceptance in LMICs may also be linked to a lack of awareness of vaccines as an effective public health intervention, known to reduce the burden of infectious diseases.^28^ Among individuals who are knowledgeable about the effectiveness and efficacy of immunisation in public health, other historical, structural, and systemic factors underpin low COVID-19 vaccine acceptance. Historically, Africa has been a victim of colonialism and medical abuse, which has reduced trust in public health interventions, including immunisation.^28,29^ For this reason, people often rely more on traditional medicine than on public health interventions.^30^ This historical context is compounded by general misinformation about vaccines and a lack of contextualised information adapted to local culture and beliefs.^31^ Furthermore, unequal global distribution of COVID-19 vaccines and long waits for vaccines have also contributed to vaccine hesitancy. Local political issues could also fuel hesitancy. In the Democratic Republic of Congo, vaccine hesitancy is linked to a lack of political trust resulting from decades of wars and outbreaks of Ebola, during which the population was unethically used for clinical trials.^24^ Similarly, low rates of vaccine acceptance were observed in the restive North-west and South-west regions in our study in Cameroon. In our study, we also observe a lack of trust in the government (597, 9.16% in Phase one and 665, 12.9% in Phase two). However, in Cameroon, factors favouring vaccine acceptance appeared to be communication with trusted sources, including health personnel. This is similar to what has been documented in a systematic review where attitudes, hesitancy and/or barriers to COVID-19 vaccine acceptability among a given population were investigated.^20^

This study identifies factors that are associated with decreased willingness of accepting the COVID-19 vaccine in Cameroon. We demonstrate that vaccine hesitancy is a context-specific phenomenon, with heterogeneity between demographic and geographical contexts. To date, very few studies have described vaccine hesitancy in Cameroon. One strength of our study is that it is a nationwide sample representative of the Cameroonian population. Knowledge about barriers to COVID-19 vaccine acceptance identified in this study conducted across Cameroon can be used to implement targeted strategies for community engagement prior to vaccination campaigns in Cameroon and elsewhere in Central Africa. Albeit these strengths, it is important to note that this study is not without limitations. This two-Phase study was cross-sectional; therefore, it was unable to establish causation given that data were collected at specific points in time. In addition, data and knowledge of vaccine efficacy, effectiveness, development and safety rapidly evolved during the study period, as did public health strategies to optimise vaccination rates. These changes might have affected the trends in vaccine hesitancy that we measured.

## Conclusion

One third of the study population were willing to receive the COVID-19 vaccine in 2021 and 2022. Acceptance of the COVID-19 vaccine varied widely across the country, with the northern part of the country having the highest proportion of respondents willing to be vaccinated. Older age, male gender, Muslim religion and low level of education were the main factors associated with vaccine acceptance. The most common concern associated with vaccine hesitancy was fear that the vaccine was dangerous. However, the Cameroonian population had a high level of trust in information provided by health workers. Therefore, vaccination and communication strategies need to be led by medical and community health workers, and should be tailored to context to ensure effective uptake, not only for COVID-19 but also for any future epidemics.
